# Prevalence of schistosomiasis and associated risk factors among school children in Um-Asher Area, Khartoum, Sudan

**DOI:** 10.1186/s13104-018-3871-y

**Published:** 2018-10-31

**Authors:** Khalid Hajissa, Abd Elhafiz M. A. Muhajir, Hamza Adam Eshag, Alnzer Alfadel, Elkhatieb Nahied, Rabeea Dahab, Safa Mohammed Ali, Marwa Mohammed, Mohamed Gaafar, Zeehaida Mohamed

**Affiliations:** 1 Department of Zoology, Faculty of Science and Technology, Omdurman Islamic University, B.O.Box382, Omdurman, Sudan; 2 Department of Medical Microbiology & Parasitology, School of Medical Sciences, Universiti Sains Malaysia, 16150 Kubang Kerian, Kelantan Malaysia; 3 Weaad Medical Center, Khartoum, Sudan

**Keywords:** *Schistosoma haematobium*, *Schistosoma mansoni*, School children, Sudan

## Abstract

**Objective:**

Schistosomiasis remains one of the most common parasitic diseases worldwide. This is a cross-sectional study aimed to determine the prevalence of schistosomiasis and its associated risk factors among primary school children in Um-Asher area. The study was conducted among 170 primary school students in Um-Asher area from November 2017 to February 2018. Urine and stool samples were collected and examined for schistosomiasis infections. Moreover, data on sociodemographic characteristics and associated risk factors were obtained using a questionnaire.

**Results:**

The overall prevalence of *Schistosoma haematobium* was 12.9%, whereas that of *Schistosoma mansoni* was 2.95%. Additionally, the males had higher prevalence (60%) of *S. mansoni* than females (40%). However, both gender were equally infected with *S. haematobium* (50%). With regard to risk factors, distance of residence from water source and source of drinking water are relatively associated with the infection.

## Introduction

Schistosomiasis is one of the most commonly neglected tropical diseases associated with significant morbidity and mortality in many developing countries in tropical and subtropical regions of Africa, Latin America and Asia [1]. Schistosomiasis remains a major health problem with significant socioeconomic impact in areas where control efforts and sanitation are inadequate and the majority of populations are impoverished [2]. The global prevalence of schistosomiasis is relatively high especially in developing countries. Recent estimates indicate that 779 million people are at the risk of infection, and 85% of them are in Africa. Approximately 207 million individuals in 74 countries are infected with schistosomiasis, and 120 million of these people developed the disease [3, 4]. Thirty-one African countries including Sudan have the great burden of this disease, and millions of individuals have been suffering from schistosomiasis [3]. Africa houses at least 90% of infected people requiring schistosomiasis treatment [5].

In Sudan, this disease is predominantly caused by two species, namely, *Schistosoma haematobium* (*S. haematobium*), which cause urinary schistosomiasis, and *Schistosoma mansoni* (*S. mansoni*), which is responsible for intestinal schistosomiasis [6]. Schistosomiasis is often associated with many factors which correlate significantly with the spread of diseases in different regions of the country. These factors include low socioeconomic status, lack of clean water supply, lack of basic infrastructure, relatively low quality of housing and poor environmental sanitation.

Schistosomiasis endemicity was first reported in Sudan in 1904 by Balfour, who investigated the presence of urinary schistosomiasis among children in Khartoum Primary School. Among these individuals, 17% were positive despite the long-time existence of the disease in this country [7–10]. Various epidemiological studies showed that infections with *S. haematobium* and *S. mansoni* are widely distributed in different regions of Sudan. Since 1919, the disease has been first reported in northern Sudan and has been prevalent in different parts of the country including Darfur (western Sudan) [11, 12], White Nile State [9] and Southern Kordofan State [13]. The disease is also endemic in different villagers of Gezira agricultural scheme [4, 14] and New Halfa agricultural scheme [10].

In developing countries, children aged 5–17 years possess the highest risk of infection and are the most infected group [15]. Personal hygiene and high frequency of contact with water in an endemic area might significantly increase the risk of acquiring this infection [3]. In addition, poor sanitation, bathing and swimming in dams and rivers or crossing rivers on the way to school barefooted, type and consistent use of toilet, uses of unprotected water sources to watering the vegetable gardens and knowledge about schistosomiasis are the risks that significantly associated with higher rates of infection [16]. The adverse effects of schistosomiasis among this group are diverse and alarming, the infection can lead to urethral and bladder fibrosis and hydronephrosis, hepatosplenomegaly, moreover, bladder cancer and colorectal cancer is a possible late-stage complication [17, 18]. Thus, this study was conducted to determine the prevalence of schistosomiasis and the associated risk factors among school age children from Um-Asher area, South Khartoum, Sudan.

## Main text

### Methodology

#### Study area

The study was conducted in Um-Asher (Al Kalakla) area located at 15°28′04ʺ North, 32°29′08ʺ East with a height of 384 m and 16.3 km south of Khartoum, Sudan.

#### Study design, period and participants

This was a cross-sectional study conducted from November 2017 until June 2018. Study participants were randomly selected from two primary schools at the study area. In these two schools, 170 children (75 males and 95 were females) were voluntary enrolled.

#### Inclusion and exclusion criteria

Children aged between 6 and 17 years attending the selected schools during the study period were enrolled. Meanwhile, all children who were seriously ill or took medication for schistosomiasis in the period of 3 weeks from the time of data collection were excluded.

#### Sample collection

All the study participants were requested to provide both urine and stool samples by using dry, clean well-labelled plastic containers. Approximately 5–10 ml of urine and 5–7 g of faeces were collected. Consequently, 10% formaldehyde was added to the stool sample as preservative, whereas no preservative material was added to the urine samples. In addition, the sociodemographic and associated risk factors were obtained via a standardized questionnaire. All samples were then transferred to the laboratory of Zoology Department for further examinations.

#### Microscopic examination

Urine samples were microscopically examined for the presence of *S. haematobium* eggs by using simple centrifugation/sedimentation [10]. The microscopic diagnosis of *S. mansoni* infection in faecal samples was performed using direct stool examination method [19]. All microscopic slides containing the eggs of *S. haematobium* or *S. mansoni* were considered positive, whereas the absence of the eggs was recorded as negative.

#### Statistical analysis

Data obtained from this study were entered into Statistical Package for Social Sciences (SPSS) version 23 and analysed using Chi square to determine the correlation between variables and risk factors. *P value* less than .05 was considered significant.

### Results

A total of 170 participants aged between 6 and 17 years from two selected primary schools in Um-Asher area were enrolled in this study. Among the participants, 95 (55.9%) were females, and 75 (44.1%) were males. Based on the age group, the majority of study participants were aged between 10 and 13 years (106 participants), whereas those aged 6–9 and 14–17 years comprised 38 and 16 of each group, respectively.

The prevalence rates of *S. haematobium* was 12.9% (22/170). The analysis indicated that males and females were equally infected (50% each). With regards to age, children aged between 10 and 13 years were the most infected with an infection rate of 81.8% compared with the other age groups. Participants who had non-sanitary latrine had higher risk for acquiring the infection (Table 1).

**Table 1:** Risk factors associated with *S. haematobium* infection (n = 170)

Variable	Infection, n (%)		χ^2^(*df*)	P value
	Positive	Negative		
Gender				
Male	11 (50%)	64 (43.2%)	.355 (1)	.551
Female	11 (50%)	84 (56.8%)		
Age				
6–9	1 (4.5%)	37 (25%)	4.747 (2)	.093
10–13	18 (81.8%)	98 (66.2%)		
14–17	3 (13.6%)	13 (8.8%)		
Distance of residence from water source				
Far	15 (68.2%)	98 (66.2%)	.33 (1)	.855
Near	7 (31.8%)	50 (33.8%)		
Type of toilet				
Sanitary latrine	8 (36.4%)	57 (38.5%)	.037 (1)	.846
Non-sanitary latrine	14 (63.6%)	91 (61.5%)		
Bathing habit				
Home	22 (100%)	129 (87.2%)	3.180 (3)	.365
Canal	0 (.0%)	3 (2.0%)		
River	0 (.0%)	3 (2.0%)		
All	0 (.0%)	13 (8.8%)		
Parents educational status				
Primary	8 (36.4%)	39 (26.4%)	1.107 (3)	.775
Inter mediate	3 (13.6%)	21 (14.2%)		
Secondary	4 (18.2%)	37 (25%)		
University	7 (31.8%)	51 (34.5%)		

The prevalence rates of *S. mansoni* were 2.9% (5/170), the infection is higher in males (60%) than in females (40%) although the difference is not statistically significant. All the infection occurred among young children up to aged 13 years old. There was no significant associated risk factors found for *Schistosoma mansoni* infection (Table 2).

**Table 2:** Risk factors associated with *S. mansoni* infection (n = 170)

Variable	Infection, n (%)		χ^2^(*df*)	P value
	Positive	Negative		
Gender				
Male	3 (60%)	72 (43.6%)	.527 (1)	.468
Female	2 (40%)	93 (56.4%)		
Age				
6–9	2 (40%)	36 (21.8%)	1.254 (2)	.534
10–13	3 (60%)	113 (68.5%)		
14–17	0 (.0%)	16 (9.7%)		
Distance of residence from water source				
Far	3 (60%)	110 (66.7%)	.097 (1)	.756
Near	2 (40%)	55 (33.3%)		
Type of toilet				
Sanitary latrine	4 (80%)	61 (37%)	3.805 (1)	.051
Non-sanitary latrine	1 (20%)	104 (63%)		
Habit of washing clothes				
Home	5 (100%)	160 (97%)	.156 (2)	.925
River and canal	0 (.0%)	2 (1.2%)		
Other	0 (.0%)	3 (1.8%)		
Bathing habit				
Home	5 (100%)	146 (88.5%)	.648 (3)	.885
Canal	0 (.0%)	3 (1.8%)		
River	0 (.0%)	3 (1.8%)		
All	0 (.0%)	13 (7.9%)		
Parents educational status				
Primary	3 (60%)	44 (26.7%)	3.015 (3)	.389
Inter mediate	0 (.0%)	24 (14.5%)		
Secondary	1 (20%)	40 (34.5%)		
University	1 (20%)	57 (34.5%)		

### Discussion

Schistosomiasis remains a public health concern in various developing countries including Sudan. School age children are among the high risk groups for *S. haematobium* and *S. mansoni* infections. The present study revealed that the prevalence of *S. haematobium* among the study participants was 12.9%, which was extremely lower comparing with 45.0%–56.0% reported from different parts of Sudan [6, 18]. However, slightly high prevalence was also reported in Gezira area, Central Sudan (20.0%) [4] and Southern Kordofan State (23.7%) [13]. In general, the high prevalence of *S. haematobium* reported among the other studies rather than this study might be due to the reason that children in developing countries including Sudan live in poor sanitation areas, and most of them often spend long time bathing or swimming in contaminated water. Additionally, hygiene and playing behaviour in contaminated water could also increase the risk of infection. However, the low infection rate reported from this study might be attributed to the small sample size variations or the mass treatment with praziquantel which established in the last few years in the study area.

In contrast to our results, high prevalence of *S. haematobium* among school age children were reported from other similar studies conducted in Ethiopia [20] and Senegal [21], wherein the prevalence rates were 20.8% and 57.6%, respectively [20, 21]. However, our finding was higher than that of the previous investigation conducted in southern Mauritania (4.0%) [5]. Additionally, an infection rate of 10.4% of *S. haematobium* among primary school children was reported in Malawi [22]. The variation in the infection rate among those areas could be due to the target population, environmental condition of the area, sample size variations and the life and behaviour of the children.

Despite the varying prevalence of *S. haematobium* between gender due to the behaviour difference that affected the rate of contact with contaminated water in most studies, unlikely, no differences were observed in the prevalence of *S. haematobium* among males and females [20]. However, males were usually more infected than females [10], and this finding could be due to the diverse outdoor activities of males exposed to cercaria-infected water. Even though, some studies indicated that the prevalence among females was higher than that among males [23]. Meanwhile, the percentage of infected children in the 10–13 year group was higher than that in the 6–9 and 14–17 year groups. This finding was similarly reported in previous studies [6, 21, 24] and might be explained by frequent contact to the river (swimming/playing) due to excessive mobility of children at this age.

On the other hand, the prevalence of *S. mansoni* was 2.9%, which is similar to that in other studies conducted previously in Sudan [18]. However, high prevalence of 51.5% of the disease was also recorded in the upper Nile region [25]. Globally, the prevalence was significantly lower than that reported in various studies conducted in Senegal (57.6%) [21], Southern Ethiopia (81.3%) [26], Congo 26.5% [27], 22.9% and 20.2% in Brazil [28]. With regard to gender, 3 out of 5 (2.9%) *S. mansoni*-infected children were males (60%) and 2 (40%) were females. This percentage is less than that reported from South Eastern Ethiopia (12.6) % [19] and the 70.9% reported from southern Kordofan [13]. The study also stated that children who live near water resources represent 60% of the infected student compared with the 40% living far from water resources. This finding might be due to the high chance of those living near the water source and playing in the water. Therefore, the results of this study emphasize the need of conducting other studies with large sample size and snail survey to assess the role of water sources in disease transmission and the implementation of effective control program to decrease the prevalence of schistosomiasis in the study area.

This study indicated the slightly high prevalence of *S. haematobium* infection and the extremely low prevalence of *S. mansoni* infection. Therefore, appropriate integrated control program and prevention measures must be implemented in the study area.

## Limitations

The study was limited by the small sample size even though it provides a preliminary data about the prevalence of schistosomiasis among school children in Um-Asher Area, however, the study still ongoing to enrol more children and schools in the area.
